# Dextran Sulfate Polymer Wafer Promotes Corneal Wound Healing

**DOI:** 10.3390/pharmaceutics13101628

**Published:** 2021-10-06

**Authors:** Remya Ammassam Veettil, Daniela C. Marcano, Xiaoyong Yuan, Mahira Zaheer, Aparna Adumbumkulath, Richard Lee, Lucas C. Isenhart, Nicole Soriano, Kirti Mhatre, Robiya Joseph, Sendurai A. Mani, Crystal S. Shin, Ghanashyam Acharya

**Affiliations:** 1 Michael E. DeBakey Department of Surgery, Baylor College of Medicine, Houston, TX 77030, USA; veettil@bcm.edu (R.A.V.); mzaheer@bcm.edu (M.Z.); aa126@rice.edu (A.A.); rl8@bcm.edu (R.L.); nicole.soriano@bcm.edu (N.S.); kirti.mhatre@bcm.edu (K.M.); 2 Department of Ophthalmology, Baylor College of Medicine, Houston, TX 77030, USA; dmarcano@bcm.edu (D.C.M.); yxy@oio.cn (X.Y.); lucas.isenhart@bcm.edu (L.C.I.); 3 Department of Translational Molecular Pathology, The University of Texas MD Anderson Cancer Center, Houston, TX 77030, USA; rjoseph7@mdanderson.org (R.J.); smani@mdanderson.org (S.A.M.)

**Keywords:** eye injury, cornea, inflammation, corneal fibrosis, corneal scarring, dextran sulfate, polymer therapeutic

## Abstract

Eye injuries due to corneal abrasions, chemical spills, penetrating wounds, and microbial infections cause corneal scarring and opacification that result in impaired vision or blindness. However, presently available eye drop formulations of anti-inflammatory and antibiotic drugs are not effective due to their rapid clearance from the ocular surface or due to drug-related side effects such as cataract formation or increased intraocular pressure. In this article, we presented the development of a dextran sulfate-based polymer wafer (DS-wafer) for the effective modulation of inflammation and fibrosis and demonstrated its efficacy in two corneal injury models: corneal abrasion mouse model and alkali induced ocular burn mouse model. The DS-wafers were fabricated by the electrospinning method. We assessed the efficacy of the DS-wafer by light microscopy, qPCR, confocal fluorescence imaging, and histopathological analysis. These studies demonstrated that the DS-wafer treatment is significantly effective in modulating corneal inflammation and fibrosis and inhibited corneal scarring and opacification compared to the unsulfated dextran-wafer treated and untreated corneas. Furthermore, these studies have demonstrated the efficacy of dextran sulfate as an anti-inflammatory and antifibrotic polymer therapeutic.

## 1. Introduction

The cornea is an outermost part of the eye and functions as a protective barrier. It is inherently devoid of blood vessels and maintains corneal transparency, which is pertinent to its primary function of transmitting light to the back of the eye [1]. Physical assaults such as abrasion, penetrating wounds, and chemical burns to the ocular surface disrupt the balance between anti-inflammatory and proinflammatory responses, triggering neovascularization in the cornea, scarring, and opacification [2,3]. If left untreated, corneal injuries can compromise corneal transparency leading to impaired vision and blindness.

The inflammatory process is triggered in response to injury, infection, and other insults, by the activated macrophages in the area of injury to initiate the recruitment of immune cells (neutrophils, monocytes, and lymphocytes). Immune cells play a crucial role during the initial inflammatory phase of the acute wound healing process by secreting cytokines such as interleukins (IL): IL-1β, IL-6, IL-8, tumor necrosis factor-α (TNF-α), monocyte chemoattractant protein-1 (MCP-1) [4,5]. In normal wound healing, the inflammatory phase is a tightly regulated process and resolves within 3–4 days, transitioning to the proliferative phase [6,7]. When the inflammatory phase fails to resolve, the injured area becomes chronically inflamed due to excessive secretion of inflammatory cytokines, delaying wound healing. Therefore, the modulation of uncontrolled inflammatory responses is essential for successful wound healing. We recently demonstrated the development of functional polymers for inflammation modulation and wound healing [8]. In this study, we present the development of sulfated polysaccharide-based electrospun wafers for inflammation modulation and promote wound healing. Numerous studies have demonstrated the potential of glycosaminoglycans (GAGs)-based biomaterials to promote wound healing by inflammation modulation [4,9,10]. GAGs are linear, high molecular weight, sulfated polysaccharides that form proteoglycans present on cell surfaces and extracellular matrices in the body [10]. GAGs are negatively charged due to the carboxylic acid and sulfate groups and electrostatically interact with the positively charged domains in the proteins [5,10]. Proinflammatory cytokines such as IL-1β, IL-6, and TNF-α are primarily basic proteins and contain heparin-binding domains that are rich in amino acids: lysine (Lys K) and arginine (Arg R) in their peptide sequences that impart positive charges [4].

Dextran sulfate (DS) is a GAG analog biopolymer containing 2–3 sulfate groups per disaccharide unit [11]. DS is negatively charged and widely used in nanoparticle and hydrogel fabrication due to its water solubility and stability. Its multiple therapeutic effects have been demonstrated [11,12,13,14,15,16,17]. DS has been shown to have a lipemea-clearing effect as clinically demonstrated to treat high blood-lipid levels [12]. It has been reported that DS showed anticoagulant activity by binding to thrombin and had inhibitory effects against scrapie virus, retroviruses, and HIV [12,15,16]. DS can also interact with the cellular membrane as it was demonstrated to prevent metastatic cancer cells from adhering to the peritoneal tissue [17]. Considering its potential therapeutic effects and physicochemical properties, we selected DS as a proof-of-concept polymer therapeutic. This strategy was to circumvent the need for exogenous anti-inflammatory drug therapies. In this study, we present the fabrication of nanofibrous DS-wafers by electrospinning method and demonstrate the anti-inflammatory and wound healing attributes of DS-wafers in mouse corneal injury models.

## 2. Materials and Methods

### 2.1. Materials

Dextran sulfate, chondroitin sulfate, dextran, Triton X-100, Whatman filter paper were obtained from Sigma Aldrich (St. Louis, MO, USA). Recombinant Mouse Cytokines, IL-1β, IL-6, and TNF-α were purchased from BioLegend (San Diego, CA, USA). Human dermal fibroblast cells, human umbilical vein endothelial cells, medium 106, medium 200, trypsin, fetal bovine serum, penicillin-streptomycin, MTT assay kit, Permount Mounting medium, goat serum, phosphate-buffered saline (PBS), Fluoromount G was obtained from Thermo Fisher Scientific (Waltham, MA, USA). Human corneal epithelial cells (CEC) and corneal epithelial cell basal medium were purchased from ATCC (Manassas, VA, USA). Human umbilical vein endothelial cells (HUVEC) and Medium 106 were purchased from Thermo Fisher Scientific (Waltham, MA, USA). Luminex assay kit (MCYTOMAG-70K) was obtained from EMD Millipore (Burlington, MA, USA). Proparacaine hydrochloride, tropicamide were purchased from Wildlife Pharmaceuticals (Windsor, CO, USA). Alcon Balanced Salt Solutions (BSS) were obtained from McKesson Medical-Surgical (Irving, TX, USA). TRIzol reagent, TURBO DNA-free kit were obtained from Ambion (Carlsbad, CA, USA). Golf Club Spud optometric instrument was from Microsurgical Technology (Redmond, WA, USA). Ready-To-Go You-Prime-First-Strand Beads were purchased from GE Healthcare (Princeton, NJ, USA), random hexamers, and TaqMan Fast Advanced Master Mix were obtained from Applied Biosystems (Thermo Fisher Scientific, Waltham, MA, USA). Paraformaldehyde, 32% was obtained from Electron Microscopy Sciences (Hatfield, PA, USA). Mouse monoclonal alpha smooth muscle actin (αSMA, SC53142) obtained from Santa Cruz Biotechnology (Santa Cruz, CA, USA) and Alexafluor 594 anti-mouse F4/80 antibody (clone BM8; Cat: 123140) obtained from Biolegend (San Diego, CA, USA). Rat anti-mouse CD31 antibody was obtained from BD Biosciences (San Jose, CA, USA). Alexa-Fluor 594-conjugated goat anti-rat secondary antibody was obtained from Jackson ImmunoResearch (West Grove, PA, USA).

### 2.2. Zeta Potential Measurement

The zeta potentials of polysaccharide macromolecules were measured using a zetasizer (Nano ZS90, Malvern Panalytical, Malvern, UK). DS and Chondroitin sulfate (CS) were dissolved in deionized (DI) water at 2.5 mg/mL concentration and transferred into a folded capillary zeta cell for the measurement. For each polymer, at least three measurements were performed.

### 2.3. In Vitro Evaluation of DS-Cytokine Interactions

Polysaccharide solutions were prepared by dissolving DS and CS in DI water at 2.5 mg/mL concentration. The macromolecule solutions were incubated with each cytokine, IL-1β, IL-6, and TNF-α in an orbital shaker at 37 °C. 1 mL each of the solutions was collected at the following timepoints: 0.5, 2, 6, 18, 24 h. The sample solutions were centrifuged in a centrifugal filter unit (MWCO: 100 kDa, Amicon Ultra, Millipore Sigma, Burlington, MA, USA).

To prepare the cytokine solution, mouse cytokine quality control-2 (MXM6070-2) from Luminex assay kit (MCYTOMAG-70K) was prepared as per the given instructions. In brief, the contents in the vial were reconstituted with 250 µL MilliQ water and thoroughly mixed. The vial was allowed to sit for 5–10 min at room temperature and then transferred to a 15 mL polypropylene tube. The cytokine solution was then constituted to 10 mL (1:40 dilution) using MilliQ water.

Mouse Cytokine/Chemokine Antibody-Immobilized Magnetic Beads for IL-1β (MIL1B-MAG), IL-6 (MCYIL6-MAG), and TNF-α (MCYTNFA-MAG), from Luminex assay kit (EMD Millipore, Burlington, MA, USA) were used for the study. The antibody beads were prepared as per the instructions given in the kit. In brief, each antibody-bead vial was sonicated for 30 s followed by vortex for 1 min. Using the assay buffer provided in the kit, 60 μL of each antibody bead solution was diluted to bring the final volume to 3.0 mL and mixed well. Each experiment was done in triplicates.

### 2.4. Fabrication of DS-Wafer

Nanofibrous DS-wafers were fabricated by electrospinning method (4SPIN electrospinning system, Contipro, Dobrou, Czech Republic). First, a 10% DS solution was prepared by dissolving 10 g of DS and 2 g of PVA in 100 mL of DI water at room temperature and transferred into a syringe connected to a multi-jet emitter via tubing. For DX, 10 g were dissolved along with 2 g of PVA in 100 mL of DI water. PVA facilitates the electrospinning of glycopolymers and provides mechanical stability to the nanofibers. The applied high voltage was 20 kV at a feed rate of 30 µL/min. The nanofibers were collected on a flat collector and carefully removed from the collector. The nanofibrous sheet (approximately 15 cm × 15 cm) was then cut into 3 mm circular DS-wafers for animal experiments. For scanning electron microscopy, DS-wafers were placed on a stub, sputter-coated with gold (Leica EM ACE 600 Sputter Coater, Leica Microsystems, Buffalo Grove, IL, USA), and imaged using a field emission scanning electron microscope at 15 keV (FEI Quanta 400 ESEM FEG, FEI, Hillsboro, OR, USA).

### 2.5. In Vitro Evaluation of DS Using Cell Models

#### 2.5.1. Cell Culture

Human corneal epithelial cells (CEC) were cultured and maintained in supplemented Corneal Epithelial Cell Basal Medium and human umbilical vein endothelial cells HUVEC were cultured and maintained Medium 200. Cells used for the study were between passages 3–6.

#### 2.5.2. Cytotoxicity Evaluation

To evaluate the cytotoxicity of glycopolymer solutions, we measured the cell viability by MTT assay. CECs were seed at ~10^4^ cells density per well in a 96-well plate. DS and CS were added to well and incubated for 24 h. Then MTT assay was performed as per the manufacturer’s technical manuals.

#### 2.5.3. HUVEC Tube Formation Assay and Flow Cytometry

HUVECs were used to evaluate the effect of DS on endothelial cells. First, Matrigel was thawed at 4 °C overnight. We then added 75 µL of cold Matrigel to each well in an 8-well chamber slide (Nunc Lab-Tek Chamber slide, system, Sigma-Aldrich, St. Louis, MO, USA), using a tip to even coat the surface. The bottom coated slide was incubated at 37 °C for 30 min prior to adding HUVECs at 3.5 × 10^4^ cells per well. Cells were incubated for 16 h to form tubular structures. We then added DS solution prepared in PBS to each well and we obtained images using an inverted microscope equipped with an environmental chamber (Nikon Eclipse Ti Microscope, Nikon Instruments, Melville, NY, USA; Okolab incubator system, Okolab, Ambridge, PA, USA). To demonstrate the DS binding to the endothelial cells, we performed flow cytometry after incubating 1 × 10^6^ HUVECs with FITC labeled DS for 30 min. Cells were rinsed with PBS and resuspended in 100 µL of PBS with 1% BSA then analyzed on an LSR II Flow Cytometer and BD FACSDiva software (BD Biosciences, San Jose, CA, USA). The fluorescence intensity of the FITC-DS treated HUVECs was compared with the untreated controls.

### 2.6. Animal Models

All animals were treated in accordance with the Association of Research in Vision and Ophthalmology (ARVO) Statement for the Use of Animals in Ophthalmic and Vision Research, and the protocols were approved by the Baylor College of Medicine Institutional Animal Care and Use Committee (AN-6573, Approval: 10/2014; AN-6382, Approval: 2/2014).

#### 2.6.1. Corneal Abrasion Mouse Model

Naive female C57BL/6 mice 6 to 8 weeks of age (The Jackson Laboratory, Bar Harbor, ME, USA) were anesthetized by intraperitoneal injection of ketamine/xylazine, combined with topical anesthesia of the right eyes by proparacaine hydrochloride (5 µL, 0.5%, topical anesthetic) and tropicamide (5 µL, 1%, mydriatic and cycloplegic agent). The central corneal epithelium was demarcated by a 2-mm trephine [18]. Then, the central epithelium was removed by softly scratching the ocular surface with a Golf Club Spud optometric instrument (MicroSurgical Technology, Accutome Inc, Malvern, PA, USA) to create the abrasion injury. The cornea was washed with sterile BSS (5 mL of balanced salt solution or BSS). The mice received once-a-day wafer treatment for 3 days. All mice then received 5 μL of BSS on the abraded cornea, including control groups.

#### 2.6.2. Ocular Burn Mouse Model

Naive female C57BL/6 mice 6 to 8 weeks of age (The Jackson Laboratory, Bar Harbor, ME, USA) were anesthetized with an intraperitoneal injection of the rodent combination anesthesia previously mentioned, combined with topical anesthesia applied on the right eyes [19]. Whatman filter paper (2.5-mm diameter) soaked in 1 N NaOH solution, was placed on the right cornea for 30 s and then the eye was immediately rinsed with 20 mL of BSS. Mice corneas were monitored daily using a stereoscopic zoom microscope (SMZ25; Nikon Instruments, Melville, NY, USA) for 10 days, and images were recorded by an SLR digital camera (Nikon Inc, Melville, NY, USA). For treatment, each day a wafer was placed on top of the ocular burn cornea of an anesthetized mouse for 10 days corresponding to the treatment group. All mice then received 5 μL of BSS on the ocular burn cornea, including uninjured (control) groups.

### 2.7. Corneal Fluorescein Staining and Imaging

The extent of corneal wound closure was examined by corneal fluorescein staining as previously described [19,20]. 24 and 48 h after corneal abrasion, mice were anesthetized with an intraperitoneal injection of the rodent combination anesthesia. A 1 μL amount of fluorescein (0.1%) was instilled on the injured corneas for 1 min, followed by rinsing with 1 mL of BSS. Images were then recorded by an SLR digital camera (Nikon Inc, Melville, NY, USA) and a stereoscopic zoom microscope (SMZ25; Nikon Instruments, Melville, NY, USA), with a fluorescence excitation at 470 nm. Three animals per group were used. Wound areas were analyzed by ImageJ software (U.S. National Institutes of Health, Bethesda, MD, USA)

### 2.8. Histology and Immunofluorescence

#### 2.8.1. H&E Staining

For H&E staining, the flash-frozen eye tissue samples were sectioned at 10 µm using a cryostat (Leica CM 1850, Leica Biosystems, Buffalo Grove, IL, USA) at −20 °C and collected on a glass slide. Five animals per group were used. The tissue section-mounted glass slides were stained with hematoxylin for 4 min and eosin for 1 min followed by dehydration and clearing in ethanol and xylene. The glass slides were then mounted using Permount Mounting medium (Thermo Fisher Scientific, Waltham, MA, USA) and sealed with a glass coverslip. The sections were imaged and analyzed using a Nikon eclipse TE2000-U microscope (Nikon Instruments, Melville, NY, USA).

#### 2.8.2. Immunofluorescence and Whole Mount Cornea

For immunofluorescence, the cryosectioned tissues were fixed with 4% paraformaldehyde for 10 min, rinsed with PBS, and blocked with 10% normal serum for 1 h. After blocking, tissues were incubated with primary antibody, either by αSMA at 4 °C overnight followed by incubation with secondary antibody in a dark chamber for 40 min at room temperature or by Alexa Fluor-594 conjugated F4/80 at 4 °C overnight. The tissues were then mounted using Fluoromount G.

Eyes were enucleated for corneal whole mount staining with some modifications [19]. Briefly, corneas including limbal area were dissected from freshly enucleated eyes, and surrounding conjunctiva, Tenon capsule, uvea, and lens were carefully removed, followed by making four slits with a scalpel blade at 90, 180, 270, and 360 to flatten out the corneas, then fixed in 4% paraformaldehyde solution at room temperature for 1 h. Tissues were blocked with 10% goat serum and 0.5% Triton X-100 prepared in PBS for 1 h. Rat anti-mouse CD31 antibody (1:300) supplemented with 5% goat serum and 0.1% Triton X-100 was added to the tissues and allowed to incubate at 4 °C for 3 days. After a series of washing with PBS and blocking with the above-mentioned solution, the tissues were incubated with Alexa-Fluor 594-conjugated goat anti-rat secondary antibody in a dark chamber for 1 h at room temperature. The tissues were then mounted on slides using Fluoromount G.

### 2.9. Confocal Imaging

Images of whole-mounted corneas were obtained by stitching individual Z-stack images (~11 × 11) acquired in a Nikon AR confocal microscope (Nikon Instruments, Melville, NY, USA) provided with a 20× objective (Plan APO20X-0.75/OFN25-DIC-N2 by Nikon) and a 561 nm laser using non-resonant Galvano scanners. Images were further processed with IMARIS 7.7.2 (Bitplane AG, Zurich, Switzerland) software for 3D representations and volume calculations. The volumes of blood vessels were calculated using the Statistic function.

Data in figures are shown as mean ± standard error of mean (SEM). Statistical significance was assessed by one-way ANOVA followed by Tukey’s test for multiple comparisons. Mean differences of the groups were considered significant at * *p* < 0.05, ** *p* < 0.01, and *** *p* < 0.001.

### 2.10. Quantification of Inflammatory Cytokines by qPCR

Mice were sacrificed at the end of the study, and eyes were enucleated. Corneas were excised and dissected from the surrounding conjunctiva and uvea. Total RNA from the cornea samples was extracted using TRIzol reagent, and stored at −80 °C. Genomic DNA was removed from the samples using TURBO DNA-free kit. RNA was quantified, and quality was assessed using Nanodrop 2000 spectrophotometer (Thermo Fisher Scientific, Waltham, MA, USA). First-strand cDNA was synthesized from 1.0 μg of RNA with Ready-To-Go You-Prime-First-Strand Beads and random hexamers. Equal amounts of synthesized cDNA were then used to measure specific gene expression by qPCR using a TaqMan Fast Advanced Master Mix for specific primers: IL-1β (Mm00434228_m1), IL-6 (Mm00446190_m1), TNF-α (Mm00443258_m1), TGF-β (Mm00436952_m1), VEGF (Mm03015193_m1), PDGFRβ (Mm00435546_m1), ACTA2 (Mm00725412_s1), CCL2 (Mm00441242_m1), CCL3/MIP1α (Mm00441259_g1), COL1a1 (Mm00801666_g1) from Applied Biosystems on Quantstudio 5 Real-time PCR system (Applied Biosystems, Thermo Fisher Scientific, Waltham, MA, USA). We used GAPDH (Mm9999915_g1) as an internal reference and the results were analyzed by the comparative threshold cycle method where target change = 2^−∆∆CT^. The results were then normalized by the CT value of GAPDH and the levels of relative expressions in the untreated, the healthy group was used as the calibrator. Three groups of five-cornea pools were processed for each group.

### 2.11. Statistical Analyses

Sample size justification for our study was performed using power analysis. Sample sizes were calculated using a formal sample size calculation. Sample sizes were calculated for all groups assuming a type I error (false positive) rate = 0.05 and power = 0.8 on a two-sided T-test. Based on preliminary data and literature data, using expected mean values and variation within groups, as well as the expected change in the means (~30% for PCR), we estimated a sample size of 5 per group. The power of the study for sample size calculation was done using a priori test of the power analysis.

## 3. Results

### 3.1. DS-Wafer Fabrication and Characterization

**Figure 1:**
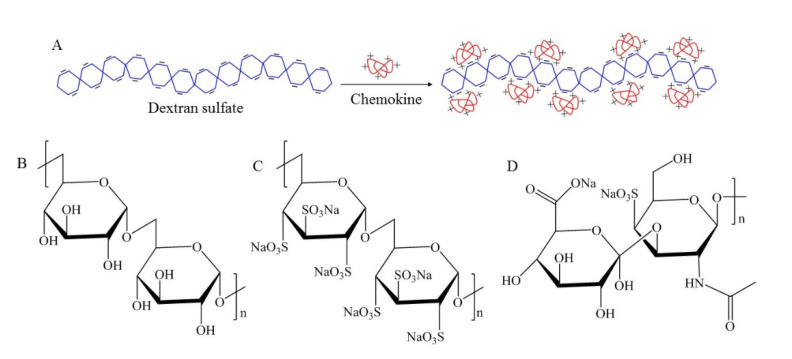
(**A**) Schematic depicting the electrostatic binding of chemokines to dextran sulfate. Chemical structures of the glycopolymers: (**B**) dextran, (**C**) dextran sulfate, and (**D**) chondroitin sulfate.

In this study, we tested three glycopolymers: Dextran sulfate (DS, MW 500,000), Chondroitin sulfate (CS, MW 1390), and Dextran (DX, MW 500,000) for their wound healing properties (Figure 1). These polymers were selected for their sulfate content, water solubility, and biocompatibility. DX is a neutral polysaccharide that has no sulfate groups [21]. DS is a synthetic sulfated glycopolymer that has two to three sulfate groups per disaccharide residue [11]. CS is an unbranched mucopolysaccharide heterodimer containing a sulfated N-acetylglucosamine or N-acetylgalactosamine and uronic acid as the two sugar units of the disaccharide residues. CS has one sulfate group per disaccharide residue. CS is one of the glycosaminoglycans (GAG) and it is present in cartilage, skin, cornea, and umbilical cord [22].

**Figure 2:**
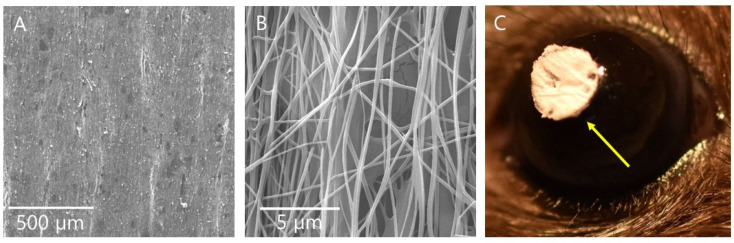
DS-wafer. (**A**,**B**) SEM images of electrospun DS-wafer. (**C**) DS-wafer applied on a mouse eye. Yellow arrow indicates DS-wafer on mouse eye.

Nanofibrous wafers of DS, CS, and DX polymers were fabricated by electrospinning method. The nanofibrous sheets were punched with a paper punch to obtain three-mm diameter circular discs of 80 µm thickness that exactly fit on mouse cornea (Figure 2). The DS-wafers are opaque and immediately adhered to the ocular surface after its application. The zeta potentials of these wafers in solution (2.5 mg/mL) were measured on a Zetasizer. The zeta potentials were DS: −44.7 mV, CS: −24.8 mV, and DX: −21.9 mV at pH 7. The zeta potential of DX was negative but higher than DS and CS as it contains no sulfate groups. The negative zeta potential value of DX arises due to the presence of OH^−^ groups present on its surface due to the equilibrium water dissociation [23].

### 3.2. DS-Wafer Binds to Proinflammatory Cytokines

**Figure 3:**
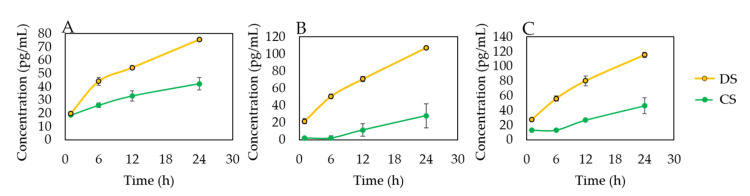
Dextran sulfate binds to the proinflammatory cytokines. Luminex multiplexed assay of (**A**) IL-1β, (**B**) IL-6, and (**C**) TNF-α. *n* = 3; All error bars represent standard deviation from the mean.

To demonstrate the cytokine binding capacity of negatively charged polysaccharides, we exposed DS and CS to proinflammatory cytokine solutions (Figure 1A). We selected proinflammatory cytokines with positively charged domains in their molecular structure: IL-1β, IL-6, and TNF-α. We incubated the glycopolymers in the cytokine solutions for a series of time points (1, 6, 12, 24 h, 1 mg/mL) followed by centrifugal filtration to remove unbound cytokines. The bound cytokines for each glycopolymer were quantified by Luminex multiplex assay. This study revealed that DS is significantly more effective in binding to IL-1β, IL-6, and TNF-α, compared to CS (Figure 3). As can be seen from Figure 3, DS with three negatively charged sulfate groups was more effective in binding to proinflammatory cytokines compared to CS molecule with a single sulfate group, thus demonstrating the importance of negatively charged sulfate groups for binding to the positively charged domains in the proinflammatory cytokines.

### 3.3. In Vitro Evaluation of DS

**Figure 4:**
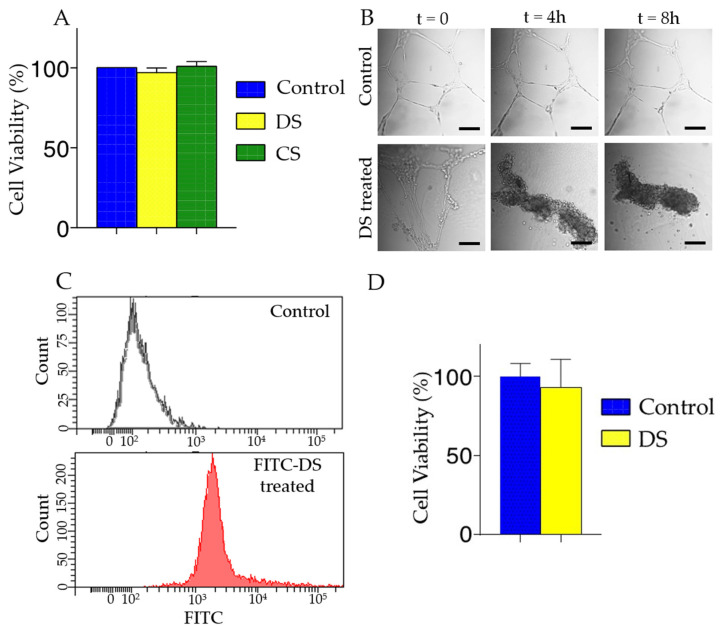
In vitro cytotoxicity and cell viability. (**A**) Evaluation in human corneal epithelial cells by MTT assay. Cells were treated with DS, CS, and PVA for 24 h. DS, CS, and PVA did not have a cytotoxic effect on human corneal epithelial cells. *n* = 6; *p* < 0.05. All error bars represent standard deviation from the mean. (**B**) Microtubular structures formed by the HUVECs are disrupted by the addition of DS. Scale bar: 250 µm (**C**) Flow cytometry profiles demonstrating the binding of DS to the HUVEC cell surface. (**D**) Evaluation in HUVECs by MTT assay. Cells were treated with DS for 24 h and showed DS treatment did not have a cytotoxic effect. *n* = 3. All error bars represent standard deviation from the mean.

To evaluate the cytotoxicity of polysaccharides, we performed MTT assay on human corneal epithelial cells treated with DS, CS, and PVA. After 24 h of incubation with polysaccharides, there was no adverse effect on cell viability compared to untreated control (Figure 4A). Next, we evaluated the effect of DS in HUVECs, a commonly used cell line to study endothelial cell functions. HUVECs cultured on matrigel formed tubes after 18 h of incubation. Once we observed tube formation, we then added DS to the chamber and imaged up to 8 h. The DS treatment disrupted tubular structures of HUVECs after 4 h causing cells to form clusters (Figure 4B), without affecting the cell viability (Figure 4D). Then we tested whether DS binds to the surface of HUVECs by flow cytometry after 30 min incubation with FITC labeled DS. As shown in Figure 4C, we observed that FITC-DS was bound to HUVEC cells.

### 3.4. DS-Wafer Promotes Corneal Wound Healing in Mouse Corneal Abrasion Model

To evaluate the efficacy of DS-wafer on corneal wound healing, we tested the DS-wafers in the mouse corneal abrasion model. Corneal abrasion is one of the most common eye injuries observed in human patients. In the mouse corneal abrasion model, the corneal epithelium is scraped off without penetrating the stromal layer [18,24]. The effect of DS-wafer on corneal epithelial migration in the abraded cornea can be monitored by fluorescence staining. The mouse corneal abrasion model enables the study of corneal surface reepithelialization and wound healing.

**Figure 5:**
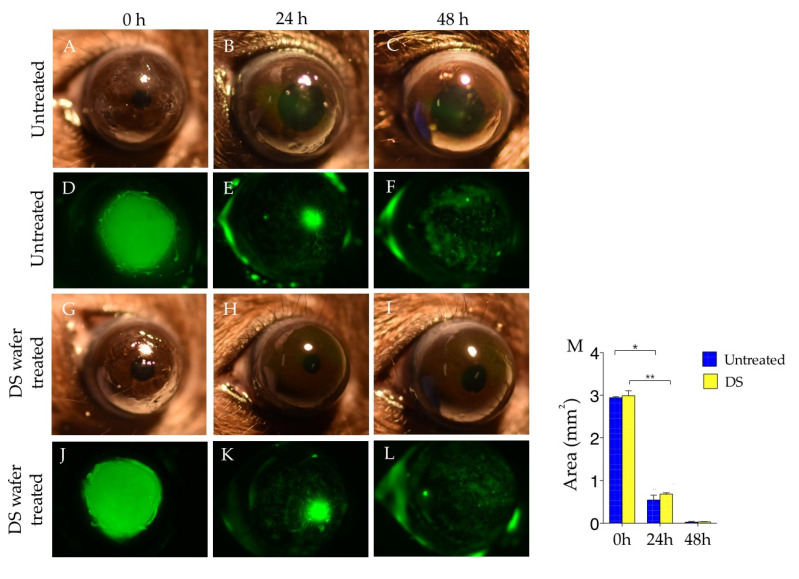
The effect of DS-wafer treatment on corneal wound healing. Corneal abrasion wound healing in untreated eyes (**A**–**C**) Bright-field images, (**D**–**F**) Fluorescence images of eyes after fluorescein staining; and in DS-wafer treated eyes: (**G**–**I**) Bright-field images, (**J**–**L**) Fluorescence images of eyes after fluorescein staining, (**M**) Wound area measurement from fluorescent images at 0, 24, 48 h after corneal abrasion. *n* = 3; * *p* < 0.05, ** *p* < 0.01; All error bars represent standard error from the mean.

The abraded corneas were treated with DS-wafers, once a day for two days. We demonstrated that the DS-wafer treatment did not interfere with the corneal reepithelialization after the abrasion since corneal reepithelialization occurs naturally after the abrasion [24,25]. Corneal wound healing was monitored by fluorescein staining followed by fluorescence imaging. The wound area was calculated using ImageJ software (Figure 5M). This study revealed that the DS-wafer treatment promoted corneal reepithelialization and there was no corneal haze or opacification compared to the untreated corneas (Figure 5).

**Figure 6:**
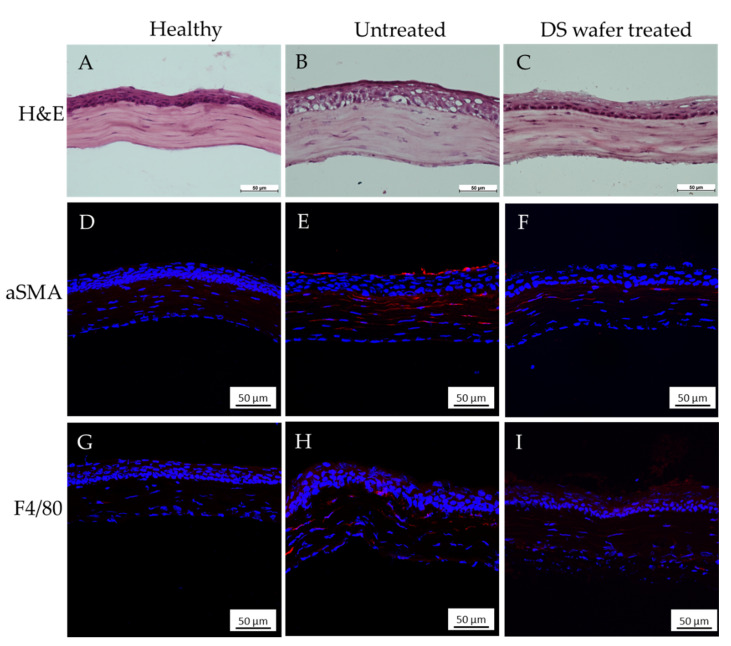
DS-wafer treatment prevents corneal fibrosis and inflammation. (**A**–**C**) H&E stained corneal cross-sections of healthy, untreated and DS-wafer treated corneas respectively. (**D**–**F**) αSMA stained corneal cross-sections of healthy, untreated and DS-wafer treated corneas respectively. (**G**–**I**) F4/80 stained corneal cross-sections of healthy, untreated and DS-wafer treated corneas respectively.

To further evaluate the corneal reepithelialization in abraded corneas, we prepared the H&E stained corneal cross-sections. A healthy cornea is smooth and has a uniformly thick epithelium and the collagen fibers in the stroma are tightly packed (Figure 6A). In abraded corneas, although the epithelium began to reform by the 2nd day, it was porous and defective, and stromal edema was observed (Figure 6B). In comparison, a three-day DS-wafer treatment (once a day) was very effective and promoted rapid reepithelialization with minimal stromal edema and a well-formed epithelium with fewer defects. The stromal collagen fibers are compact and tightly packed compared to the untreated cornea (Figure 6C).

To evaluate corneal fibrosis, we stained the corneal sections with α-smooth muscle actin (αSMA) to assess the myofibroblasts in the corneal stroma. During the corneal wound healing, the αSMA positive myofibroblasts stimulate fibrosis and cause corneal scar formation [26]. After treating the abraded corneas daily with DS-wafers for three days, the corneal sections were immunostained with αSMA antibody. The immunostained corneas have revealed that, in untreated corneas, there is a higher expression of αSMA (red fluorescent areas) in the corneal epithelium and also in the stromal layer indicating the presence of myofibroblasts compared to a healthy corneal section wherein the myofibroblasts are entirely absent. The DS-wafer treated corneal sections showed a negligible presence of myofibroblasts compared to untreated, injured corneas (Figure 6D–F). This study has indicated that DS-wafer treatment reduces αSMA positive myofibroblast transformation and thus prevents corneal scarring.

To assess the level of inflammation as a measure of the presence of immune cells in the abraded corneas, the corneal sections were immunostained with F4/80. Fluorescence imaging of the F4/80 stained corneal sections revealed a reduced level of immune cell invasion in DS-wafer treated corneas compared to the BSS-treated corneas. These studies demonstrated that a three-day DS-wafer treatment promoted corneal reepithelialization and corneal wound healing with reduced scarring in comparison to the untreated corneas.

**Figure 7:**
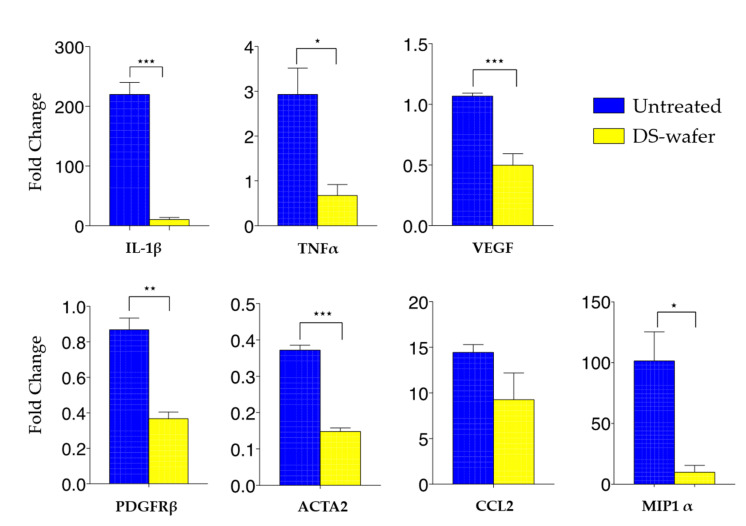
DS-wafer treatment downregulates the proinflammatory cytokines and profibrotic factors in the injured cornea. qPCR analysis of the expression levels of proinflammatory, and profibrotic factors in mouse corneal abrasion model after three days of DS-wafer treatment. *n* = 3 groups (5 corneas per group). * *p* < 0.05, ** *p* < 0.01, *** *p* < 0.001). All error bars represent standard deviation from the mean.

To further evaluate the anti-inflammatory and antifibrotic properties of the DS-wafer at the molecular level, we conducted a qPCR analysis of the corneas to quantify the expression levels of proinflammatory cytokines and profibrotic factors in the cornea. In response to corneal injury, there will be a rapid invasion of immune cells (macrophages, monocytes, neutrophils, etc.) to the injury site. Macrophages are the key regulators of the inflammatory process. During the acute phase of the inflammation, macrophages secrete proinflammatory tumor necrosis factor-α (TNF–α), interleukin-6 (IL-6), and macrophage inflammatory protein-1α (MIP-1α). TNF–α plays a key role in perpetuating inflammation by upregulating other proinflammatory cytokines and endothelial adhesion molecules which stimulate the recruitment of leukocytes to the site of inflammation. The MIP-1α is a chemokine that perpetuates the acute inflammatory condition by recruiting/activating polymorphonuclear leukocytes (PMN) [3,27]. To investigate the role of proinflammatory cytokines and proangiogenic factors in inducing inflammation and fibrosis, the corneas were subjected to qPCR analysis. This study demonstrated that the DS-wafer treatment downregulated the proinflammatory cytokines IL-1β, MIP1, and TNF-α, and profibrotic ACTA2, PDGFR-a, CCL2, and VEGF (Figure 7). Taken together, these studies have demonstrated the anti-inflammatory and antifibrotic properties of the DS-wafer in a mouse corneal abrasion model.

### 3.5. DS Wafer Prevents Corneal Scarring and Opacification

**Figure 8:**
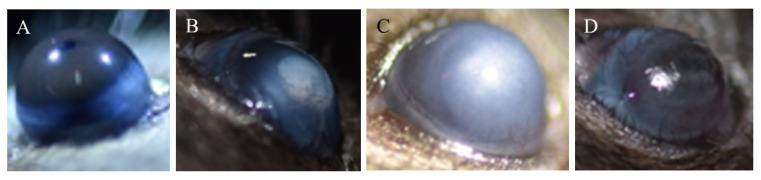
Dextran sulfate wafer is effective in minimizing corneal scarring in ocular burn-induced mouse model. Light microscopy images of (**A**) healthy eye, (**B**) OB-induced eye, (**C**) DX-wafer treated, and (**D**) DS-wafer treated eye.

To evaluate the anti-inflammatory and antiangiogenic properties of DS-wafer and its ability to prevent corneal scarring, we applied the DS-wafers on the corneas of ocular burn (OB) induced mouse model. In the OB mouse model, slight exposure of cornea to mild alkali (NaOH) solution triggers damage to the corneal epithelium and causes extensive corneal neovascularization (CNV) and scarring [19,26]. The OB mouse model presents an aggressive corneal injury wherein the corneal epithelium is chemically dissolved by exposure to mild alkali that triggers rapid neovascularization and corneal scarring. The OB-induced mice were treated with DS-wafers, once a day for 10 days followed by light microscopy imaging. DS-wafer treatment resulted in a relatively smooth and transparent corneal surface with minimal corneal scarring and opacification. In comparison, DX wafer treated eyes were white and highly opaque similar to the untreated OB group (Figure 8). In our experiments, we consistently observed that the CS and DX wafer treatments caused a high inflammation and suffering of the animal. Hence, we discontinued further testing with DX and CS wafers.

**Figure 9:**
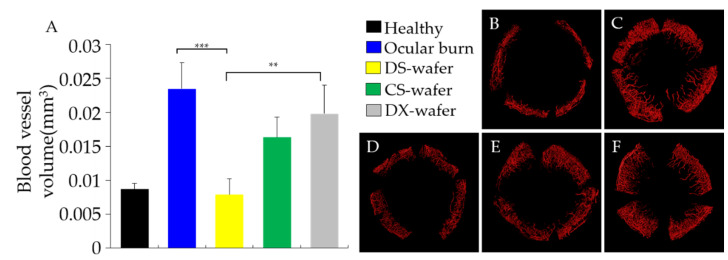
DS-wafer is effective in inhibiting corneal neovascularization. (**A**) Quantification of corneal blood vessel volumes. (**B**–**F**) Representative 3D reconstructed confocal images of whole-mount corneas stained with CD31 antibodies. (**B**) Normal, healthy cornea; (**C**) OB-induced, untreated cornea; (**D**) DS-wafer treated cornea; (**E**) CS-wafer treated cornea; (**F**) DX-wafer treated cornea. *n* = 3, ** *p* < 0.01, *** *p* < 0.001. All error bars represent standard deviation from the mean.

In this study, DS-wafer treatment was compared with CS-wafer and DX-wafer on inhibiting CNV. The mice received wafer treatment once a day for 10 days. A circular DS-wafer was placed on the injured cornea under general anesthesia, daily for 10 days. On the 11th day, the corneas were collected and processed to obtain whole-mount corneas. The vascular endothelium in the corneas was labeled with CD31 fluorescent marker for laser confocal fluorescence imaging (Figure 9). This study revealed that the blood vessels in the healthy wild type (WT) mouse cornea are present only in the limbal area and the rest of the cornea is avascular (Figure 9B). However, soon after the OB injury, new blood vessels developed from the limbal area and rapidly proliferated towards the center of the cornea resulting in CNV (Figure 9C). The CNV is highly branched and intricate. Once a day DS-wafer treatment inhibited the CNV formation and restricted it to the limbal area (Figure 9D). Lowly sulfated CS-wafer treatment has a mild inhibitory effect on CNV (Figure 9E). In the case of unsulfated DX-wafer treated corneas, the CNV density was close to the untreated OB control group and has no effect on the inhibition of CNV formation (Figure 9F).

To quantify the DS-wafer therapeutic efficacy as a measure of CNV density, whole-mount confocal fluorescence images of the corneas were analyzed by IMARIS software. As can be seen from Figure 9A, DS-wafer treatment was more effective in inhibiting CNV in OB-induced mice compared to lowly sulfated CS-wafer and unsulfated DX-wafer treatments. DS-wafer treatment has restored CNV levels comparable to the healthy corneas. On the other hand, unsulfated DX-wafer treatment has no effect on CNV inhibition and the CNV density is very close to that of the untreated OB group. Taken together, these results demonstrated that the DS-wafer was twice as effective in inhibiting the CNV compared to the untreated control and unsulfated DX-wafer treated groups.

**Figure 10:**
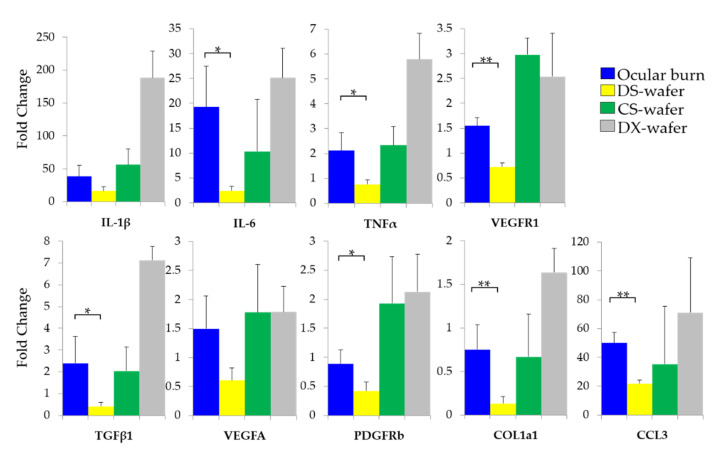
Enhanced therapeutic effect of DS-wafer. qPCR analysis revealing the strong suppression of the expression levels of target genes by DS-wafer compared to DX-wafer and CS-wafer treatment. *n* = 3 groups (5 corneas per group). All error bars represent standard deviation from the mean. * *p* < 0.05, ** *p* < 0.01.

To further evaluate the antiangiogenic and antifibrotic attributes of the DS-wafer treatment at the molecular level, a qPCR analysis of the corneal tissue was performed. The DS-wafers were instilled on the corneas of OB-induced mice, daily for 5 days. At the end of the treatment period, the corneas were collected and processed for evaluating proangiogenic and profibrotic factors by qPCR analysis. The expression levels of cytokines and growth factors were normalized to GAPDH and the levels of relative expressions in the untreated, healthy group were used as the calibrator. In Figure 10, the results were presented as relative fold changes. This study revealed that the DS-wafer downregulated the expression levels of proinflammatory IL-6, MIP-1α (CCL3), and TNF-α compared to the untreated OB corneas. The expression levels of IL-6, MIP-1α, and TNF-α are significantly lower in DS-wafer treated corneas than in untreated OB corneas. To evaluate the antiangiogenic effect of DS-wafers, the expressions levels of proangiogenic growth factors: VEGFR1 and PDGFR-β were quantified. The DS-wafer treatment has significantly downregulated the VEGFR1 and PDGFR-β expression levels. In addition, the DS-wafer treatment also downregulated profibrotic TGF-β and collagen type I-α1 (COL1a1) expression levels. Taken together, DS-wafer is significantly effective in down-regulating the proinflammatory cytokines, proangiogenic growth factors, and profibrotic factor expression levels compared to the untreated OB corneas.

## 4. Discussion

Polymer therapeutics encompass an evolving class of functional polymers that are devoid of low molecular-weight pharmacological drugs and associated toxic side effects. Polymer therapeutics are multivalent because of the presence of repeating monomer units. Multivalency of the polymer therapeutic enables it to simultaneously bind to several cell surface receptors or target molecules compared to monovalent small molecular drugs. Therapeutic efficacy and multivalency of the polymer therapeutics can be modulated by its molecular weight and the number of monomeric units without affecting its safety or effectiveness. Thus, polymer therapeutics can provide an effective alternative to small molecular drugs. The use of GAG or polysaccharides-based polymers have been explored as wound dressing to promote skin wound healing because of their well-characterized biocompatibility and ease of chemical and physical modifications [11,28,29]. Considering skin and cornea share similar physiological functions and biological composition, naturally, these polymers have been used to develop as ophthalmic delivery systems or incorporated as formulations [30,31]. The use of polymer as therapeutics to treat corneal injuries has been limited. However, a recent study involving a non-toxic glucosamine derivatives showed enhanced corneal healing followed by alkali burn in rabbits [32]. GAG or polysaccharides-based polymers such as hyaluronic acid, dextran, chitosan, are used in ophthalmic applications as eye drops. The eye drop formulation although easy to apply is not efficient since the majority of the solution applied is lost due to the blinking and drainage. The DS-wafer application minimizes the loss and increases the efficiency by reducing the frequency of applications.

Ocular trauma elicits a strong inflammatory reaction and a dysregulated inflammatory process, which can prolong the secretion of positively charged proinflammatory cytokines such as IL-1β, IL-6, and TNF-α [19,26]. IL-1β is secreted by macrophages and IL-6 stimulates the inflammatory response and is mainly produced by macrophages, monocytes, fibroblasts, and endothelial cells [27,33,34]. Another proinflammatory cytokine, TNF-α, is produced by activated macrophage and monocytes [35]. We were interested in these cytokines as they contain positively charged amino acids such as arginine (Arg; pKa 12.48) and lysine (Lys; pKa 10.79) in their peptide sequence or heparin-binding domain. IL-1β is positively charged because it contains the Lys (K) rich polybasic domains in the peptide sequences, KQYPKKK and KLILKKK [27]. In IL-6, Arg-rich amino acid sequences impart a positive charge [33,34]. TNF-α contains a positively charged heparin-binding domain [35].

The isoelectric point pH of Arg and Lys are 10.76 and 9.74, respectively. Since the ocular surface pH is in the range of 7–7.4, Arg and Lys present in IL-1β and IL-6 will remain positively charged on the ocular surface [27,33,34]. Therefore, we hypothesized that the negatively charged DS could electrostatically bind to the positively charged domains of the proinflammatory cytokines and sequester them from the corneal injury site to modulate local inflammation, preventing corneal scarring and opacification.

In this work, we demonstrated the use of DS-wafer as a drug-free polymer therapeutic to promote wound healing. The presence of negatively charged sulfate groups on the disaccharide units of the DS polymer chain imparts it with multivalency. Injury to the cornea due to abrasion, alkali burn, or prolonged inflammation triggers the production of proinflammatory and proangiogenic factors causing neovascularization, corneal scarring, and loss of sight. Hence, we evaluated the DS’s ability to sequester the excess proinflammatory cytokines, and proangiogenic and profibrotic factors to prevent corneal neovascularization and corneal opacification in cornea injury mouse models.

Our studies revealed that, at the macroscopic level, DS-wafer treatment inhibited corneal opacification and minimized corneal neovascularization after severe ocular alkali burn injury. The DS-wafer treated corneas were smooth and transparent compared to DX-wafer or untreated OB corneas. At the molecular level, the DS-wafer treatment was very effective in downregulating the expression levels of proinflammatory IL-6, TNF–α, and MIP-1α, and proangiogenic VEGF-R1, PDGFR-β, TGF-β, and COL1A1 expression levels in corneal tissues compared to untreated, injured corneas. On the other hand, neutral DX-wafer treated corneas expressed the proinflammatory and proangiogenic factor levels comparable to the untreated OB corneas.

We focused on the effect of DS on proinflammatory cytokines in this study because the stimulated proinflammatory cytokines eventually lead to the neovascularization in injured corneas. In healthy cornea, angiogenic and immune privilege is maintained by balancing the proinflammatory and anti-inflammatory cytokines [36]. As we observed that expressions levels of VEGFa, PDGFR-β, and COL1A1 from DS-wafer treated corneas were lower than the healthy, untreated group, this may be the reason why the corneal neovascularization was prevented in injured, DS-wafer treated corneas. Although more comprehensive investigation to reveal the exact mechanism of corneal wound healing by cytokine sequestration, in our study, we showed that the negatively charged DS-wafer treatment was effective in promoting corneal wound healing without using pharmaceutical agents. The combined effect of anti-inflammatory and antiangiogenic attributes of the DS-wafer was manifested in the scar-less healing of the corneas after severe injury.

In summary, the results presented here demonstrate the enhanced therapeutic efficacy of DS-wafer as a drug-free polymer therapeutic for preventing corneal neovascularization and corneal scarring and promote the wound healing process.

## Data Availability

The data presented in this study are available on request from the corresponding authors.
